# Bridging the Gap: Exploring Opportunities, Challenges, and Problems in Integrating Assistive Technologies, Robotics, and Automated Machines into the *Health Domain*

**DOI:** 10.3390/healthcare11172462

**Published:** 2023-09-04

**Authors:** Daniele Giansanti

**Affiliations:** National Centre for Innovative Technologies in Public Health, Italian National Institute of Health, 00161 Rome, Italy; daniele.giansanti@iss.it

The field of healthcare is continually evolving and advancing due to new technologies and innovations. Assistive technologies (ATs), robots, and automated machines have emerged as powerful tools in the *health domain*, capable of revolutionizing overall care [1]. These technologies have the potential to transform the delivery of healthcare, and range from wearable devices for physiological parameter monitoring up to rehabilitation robots assisting with and supporting rehabilitation processes. According to the World Health Organization (WHO), ATs can improve both participation and inclusion, particularly for individuals with disabilities and frailties [2,3]. Their primary purpose is to preserve or enhance an individual’s functioning and independence, enabling them to engage in all aspects of life. Care robots (CRs) offer various interesting applications that can range from rehabilitation up to surgeries [4,5,6]. Automated machines are permeating the health system in various fields [7,8]. They comprehend artificial intelligence (AI)-based systems that can be integrated in the hardware and software and, more generally, in the interoperable systems of the *health domain* [9]. As a result, the perspective of care in the health domain is undergoing radical changes, with the potential to transform medicine.

ATs, CRs, and automated machines (AMs) are strategic in the *health domain*. To explore the opportunities, challenges, issues, and bottlenecks of integrating these tools (alone or interconnected) into the domain, this Special Issue [10] was initiated in May 2022, and was subsequently completed in January 2023. This Special Issue collected 17 contributions plus an editorial [1,11,12,13,14,15,16,17,18,19,20,21,22,23,24,25,26,27], and covered a wide range of topics, including:

- Portable and wearable devices for remote patient monitoring and home-based healthcare;
- Robotics in healthcare;
- Automated machines for diagnosis, imaging, and service automation in the health domain;
- Artificial intelligence and machine learning in the health domain;
- Ethical and regulatory considerations in the use of these technologies;
- Applications of virtual reality in the health domain;
- Integration into digital health and telehealth.

A detailed analysis of the issues addressed is briefly summarized to better highlight these fields with more details.

Strunga et al. [11] overviewed the contemporary applications of advanced AI software in orthodontics, showing both the opportunities and the problems of integration in the *health domain*.

Kim et al. [12] investigated enhancing usability and satisfaction among adults in community living through functional and gait exercises utilizing a wearable hip exoskeleton. They showed that a single session of exercise with this tool allowed an improvement in performance of both middle- and old-aged adults.

Ianculescu et al. [13] proposed and successfully applied a method, comprising remote monitoring digital solutions, for identifying and managing the risk of long-term emotional and mental declines in older adults following a SARS-CoV-2 infection.

Ha et al. [14] explored the impact of a stroke rehabilitation method that employs gait-robot-assisted training and tailored tasks on motion, daily activities, self-efficacy in stroke recovery, and overall quality of life among patients with hemiplegia post-stroke. The study highlighted that the integration of gait-robot-assisted rehabilitation and goal setting notably enhanced gait proficiency, balance, self-assurance in stroke management, and the overall quality of life for stroke patients.

Hu et al. [15] designed equipment dedicated both to urine and stool management in disabled persons. The study showed that the system could improve the efficacy of conventional detection approaches; furthermore, the developed excretion nursing equipment successfully achieved the task of providing excretion care for patients.

Zhu et al. [16] empirically investigated the role of faith, privacy fears, and observed helpfulness in client approval and ongoing intention in telehealth. The findings indicated that faith influenced the connection between subjective helpfulness and approval with ongoing intention. Privacy fears were found to potentially moderate the association between faith based on structural assurance and ongoing intention.

Ju et al. [17] analyzed the design, functioning, and outcome of different robotic rehabilitation systems in the field of sports. The study highlighted the potential use of these systems in elderly patients with degenerative pathologies.

Pirrera et al. [18] conducted an overview of Ats, using devices based on the control of the tongue with an arranged barbell piercing. They showed that these smart devices were promising, and suggested better integration into the *health domain*.

Leung et al. [19], assuming that social robots had the potential to bring benefits to aged care, investigated the effectiveness of human–robot interactions. They concluded that, in order to support aging-in-place and fill the gaps of the intensified shortage of health and social manpower, it was of prime importance to develop reliable, age-friendly, AI-based robotic services meeting the expectances and desires of older adults and caregivers. Samee et al. [20] focused on medical devices used for computer-aided diagnoses. Their study proposed a novel deep convolutional neural network for the classification of brain cancer in images obtained by means of magnetic resonance systems. Overall, the results showed an improved accuracy compared to prior studies.

Kavalieros et al. [21] proposed a procedure for choosing suitable mechatronics for robotic devices designed for the lower limbs. The outcome demonstrated the correctness and effectiveness of the proposed procedure.

Kalafati et al. [22] reported the performance of a novel prototypal application for smartphones. It used a compression pen, with the role of collecting measurable and unbiased information on patients with Parkinson’s Disease (PD), with the potential of allowing the physicians to better face and classify the degree of severity of the disease.

Giansanti D. commented in [23] on the study by Kalafati et al. [22], discussing the important perspectives of the tool to pave the way for the application of self-assessments by means of mHealth in PD.

Jin et al. [24] assumed that children in the health domain face a lot of stressful circumstances, and that robots may create fun and friendly environments for children. They investigated the desirable requirements of care robots for producing outcomes, which contributes to highlighting the importance of the robots for assisting patients of pediatric age in the health domain in the future.

Giansanti [25] focused on AI integration in digital radiology, and highlighted the complexity and intricacy of the adoption of regulation in this field both at a national and an international level.

Hanna et al. [26], in a study conducted in Texas, explored the application of social networks as ATs in the field of epidemiology. They investigated the cost trends of advertisements on Facebook as a function of new HIV diagnoses with different models of communication approach. The study revealed that, for populations at risk of HIV, directed Facebook adverts proved more cost-effective for identifying new HIV infections compared to non-directed advertisements.

Tokgöz et al. [27] provided a review of the area of virtual reality for upper-extremity rehabilitation. Their results emphasized the need for stronger evidence-based virtual-reality technologies for the rehabilitation of injuries and diseases of the upper extremities.

From this brief detailed examination, this collection is shown to highlight the following:

- The important interest of scholars in the field of robotics, both as regards rehabilitation aspects [12,14,17] (also through protocols that simulate sports activities [17]), and in the applications of social robotics [19,24], and as regards technological-propulsive aspects [21];
- The need for more scientific evidence in the effectiveness of using virtual reality [27];
- The criticality of regulatory and ethical aspects in these highly innovative technological systems, both with and without AI content [18,25];
- The interest in topics on digital health and telehealth, whether integrated with AI (as, for example, in a study on a software for orthodontics [11]) or not, for web-based psychological studies on the elderly [13], for studies on the factors influencing their use [16], and for studies focused on innovative applications in PD [22], with interesting prospects for self-assessment [23];
- AMs have found application in innovative machines called *excretion nursing equipment* [15] within smart medical devices [18], and in innovative feature recognition algorithms in BT [20];
- Even social networks, in this case Facebook, have found space as applications in the *health domain* [26].

It is also useful to make a mapping point, through this Editorial, on trends in this sector, to verify and possibly underline and reinforce the interest of a global scientific focus on this sector, which pushes us to move forward in initiatives towards the collection of experiences, such as Special Issues.

As regards the studies on ATs, a search with the key reported in *Box 1*, *position* 1 highlights 2803 studies starting from 1990. Of these studies, 966 (34.5%) were carried out starting from 1 January 2020. In all, there are 428 reviews (both systematic and non-systematic).

As regards the studies on robotics, the search with the key reported in *Box 1*, *position 2* highlights 12,868 studies starting from 1978. Of these studies, 5827 (45.3%) were carried out starting from 1 January 2020. In all, there are 2671 reviews (both systematic and non-systematic).

As regards the studies on automated machines, the search with the key reported in *Box 1*, *position 3* highlights 488 studies starting from 1968. Of these studies, 330 (67.6%) were carried out starting from 1 January 2020. In all, there are 15 reviews (both systematic and non-systematic). As far as automated machines are concerned, it is also useful to search using other keys (e.g., neural networks, machine learning, deep learning, artificial intelligence). Limiting ourselves to research on AI, we can see how, as regards the studies on AI, the search with the key reported in *Box 1*, *position 4* highlights 35,542 studies starting from 1969. Of these studies, 29,608 (83.3%) were carried out starting from 1 January 2020. In all, there are 8801 reviews (both systematic and non-systematic).

This brief overview highlights how, in these sectors, there has been a tremendous acceleration of scientific production and interest in the period of the COVID-19 pandemic. The interest in studies on automated machines is older than the others (1968). There is a good percentage of reviews (also considering the AI inside automatic machines), indicating good progress in the stabilization process of topics of scientific interest.

Box 1 **Composite key used for the searches in PubMed.**

*(assistive technology[Title/Abstract])*

*(robotics[Title/Abstract])*

*(automated machine[Title/Abstract])*

*(artificial intelligence[Title/Abstract])*

*Overall*, this Editorial highlighted the interest of scholars in ATs [28,29,30,31], CRs [32,33,34,35], and AMs [36,37,38]. This interest has increased, becoming tremendous in the last three years, due to the COVID-19 pandemic.

There is a great and renewed need for discussion in this area, in order to exchange and share experiences at 360 degrees on opportunities, problems, and even failures. With this in mind, this Special Issue, “Assistive Technologies, Robotics, and Automated Machines in the Health Domain: Second Edition” [39] was launched.
